# Ethnicity and the ethics of data linkage

**DOI:** 10.1186/1471-2458-7-318

**Published:** 2007-11-08

**Authors:** Kenneth M Boyd

**Affiliations:** 1 College of Medicine and Veterinary Medicine, University of Edinburgh. Biomedical Teaching Organisation, Medical School, Teviot Place, Edinburgh EH8 9AG, UK

## Abstract

Linking health data with census data on ethnicity has potential benefits for the health of ethnic minority groups. Ethical objections to linking these data however include concerns about informed consent and the possibility of the findings being misused against the interests of ethnic minority groups. While consent concerns may be allayed by procedures to safeguard anonymity and respect privacy, robust procedures to demonstrate public approval of data linkage also need to be devised. The possibility of findings being misused against the interests of ethnic minority groups may be diminished by informed and open public discussion in mature democracies, but remain a concern in the international context.

## 

Health services are required to demonstrate that they are meeting the needs of ethnic minority populations. This is difficult, because routine data on health rarely include reliable data on ethnicity. But data on ethnicity are included in census returns, and if health and census data for the same individuals can be linked, the problem might be solved. These conditions apply in Scotland, where an innovative study, linking the health and ethnic data of 4.6 million people, has uncovered important information about the incidence of, and survival after, acute myocardial infarction among South Asians [1]. Linkage was achieved by techniques, developed to protect the anonymity of the individuals involved, which ensured compliance with data protection legislation and received appropriate regulatory approval. This pioneering study paves the way for further work which could have considerable importance for health service planning and, its authors suggest, potentially has international applicability. The benefits of linking health and ethnic data could be great. But are there also harms? Two ethical objections to data linkage need to be answered.

The first objection concerns informed consent. Individuals who stated their ethnicity in census returns were not told that this might subsequently be linked with their health data. Their consent to providing this personal data thus was not fully informed, and so it cannot be relied upon as valid consent. The only ethically correct course is to return to each of these individuals and seek explicit informed consent for this specific use of their personal data.

Given the impracticability of this requirement, it may be tempting to dismiss this objection as ethically disproportionate: the potential benefits of data linkage surely vastly outweigh the merits of such moral scruples. But not all official uses of personal information about citizens are potentially innocent, and overruling the principle of informed consent is morally hazardous. Whether that principle always requires explicit and specific consent to every possible use of an individual's personal data may be questioned however. Philosophers Neil Manson and Onora O'Neill, for example, argue that the ideal of fully explicit and completely specific informed consent in fact is unachievable. The idea that certain types of information can be fenced off as 'personal' and protected by consent requirements, moreover, overlooks the possibility that much of this information may already be known to, or readily be inferred by, others [2]. What matters morally, they suggest, is not so much that such information is 'personal', as how it is acquired and communicated, and whether in the process the individual's right to privacy is respected. Others may already know or infer, for example, some of the personal information that a patient communicates to a doctor. But none of this information, if acquired in a confidential patient-doctor relationship, may be communicated by the doctor to others without the patient's consent, or unless the others have a need to know or, rarely, the doctor is under a legal requirement to disclose certain information.

Respect for the individual's right to privacy is no less central to the ethics of how information provided in a census return or for health statistics is acquired and communicated. But this right can be respected, Manson and O'Neill argue, by procedures which 'make information *effectively *anonymous to those who do not need to know the identity of a data subject' [2]. This requirement appears to have been met by the encryption methods and organisational procedures carefully crafted for the record linking in Scotland: neither of the organisations involved in the study – the NHS Information Services Division and the General Register Office – could view the other organisation's primary datasets (the census data and the hospital discharge/death linked data) in a form in which individuals were identifiable' [1].

If Manson and O'Neill are right, this sounds reassuring. But the arguments of philosophers can always be contested and the assurances of officials are not always trusted. Further reassurance is offered by the authors of the Scottish study when they say that their work was supported by bodies such as the Commission for Racial Equality, and that they 'disseminated information widely to encourage people to discuss the proposal and the early findings' [1]. Informed public discussion clearly must play a vital part in deciding public policy on data linking, not least because, *pace *Manson and O'Neill, their interpretation of informed consent requirements remains controversial [3]. But whether simply disseminating information and encouraging discussion is sufficient for this purpose is doubtful. If the use of record linking without first seeking individual informed consent is to proceed with public confidence, more robust procedures may be needed to provide evidence that the public in general and ethnic minority populations in particular not only have participated in fully informed discussion of the issues, but also that these discussions have led to positive approval of what is proposed.

The need for public participation and positive approval is strengthened by consideration of the second ethical objection to data linkage. This is the possibility that public information or misinformation derived from the findings of linked data studies could be used to stigmatise, coerce or physically harm an ethnic minority group. An ethnic minority group might be discovered, for example, to have an unusually high incidence of, or predisposition to, an inheritable condition deemed undesirable by or harmful to the majority population. In extreme political circumstances the minority group might come under pressure or even coercion not to reproduce. In less extreme circumstances there might be populist stigmatisation of the group. Such possibilities, of course, remain theoretical. But the best defence against their occurring is in the quality of democratic debate and public participation in decision-making. A society whose discussion of public policy is informed and mature, and in which all shades of public opinion, by openly engaging with one another, come to acknowledge the moral complexity of the issues involved, leaves less room for demagogues or dictators to operate.

With this in mind, some caution may need to be exercised with respect to the authors' view that their approach potentially has 'international applicability'. Not all countries are democracies, and some have adopted coercive population control policies. It is not impossible either that some might again, as in the 20^th ^century, decide to adopt eugenic policies. In the international context, the second ethical objection to data linkage cannot be dismissed. The risk remains that linkage methods developed, with proper safeguards, for just and beneficent reasons, could be employed elsewhere, without these safeguards, for ends that are unjust and maleficent. This risk again of course must be set against the considerable potential benefits of data linkage to the health of ethnic minority populations. Here, as in many other spheres of life, the general ethical challenge is to pursue what is good, while avoiding what is wrong, recognising that this calls for a creative response to moral complexity. The recent Scottish initiative certainly is to be welcomed. But if further work of this kind is to proceed, robust procedures for monitoring its ethical aspects and ensuring positive public approval need to be devised. This will be particularly challenging in the international context, where definitions of ethnicity are controversial, public health programmes may not be delivered by a single national health service, and questions arise about who will monitor such studies, or who will be the 'public' whose approval is to be ensured.

## Competing interests

KB declares that he has no competing financial interests. KB is an academic colleague (in the University of Edinburgh) of one the authors of the paper which is the subject of the Commentary.

## Pre-publication history

The pre-publication history for this paper can be accessed here:



## References

[B1] Fischbacher CM, Bhopal R, Povey C, Steiner M, Chalmers J, Mueller G, Jamieson J, Knowles D (2007). Record linked retrospective cohort study of 4.6 million people exploring ethnic variations in disease: myocardial infarction in South Asians. BMC Public Health.

[B2] Manson NC, O'Neill O (2007). Rethinking Informed Consent in Bioethics.

[B3] Wilson J (2007). Is respect for autonomy defensible?. J Med Ethics.

